# Exploiting Saturation Regimes and Surface Effects to Tune Composite Design: Single Platelet Nanocomposites of Peptoid Nanosheets and CaCO_3_

**DOI:** 10.1021/acsami.4c00434

**Published:** 2024-04-03

**Authors:** Seniz Ucar, Anne R. Nielsen, Biljana Mojsoska, Knud Dideriksen, Jens-Petter Andreassen, Ronald N. Zuckermann, Karina K. Sand

**Affiliations:** † Department of Chemical Engineering, Norwegian University of Science and Technology, Trondheim 7491, Norway; ‡ Department of Metallurgical and Materials Engineering, Middle East Technical University, Ankara 06800, Turkiye; § Nano-Science Center, Department of Chemistry, University of Copenhagen, Copenhagen 2100, Denmark; ∥ Department of Science and Environment, Roskilde University, Roskilde 4000, Denmark; ⊥ Biological Nanostructures Facility, The Molecular Foundry, Lawrence Berkeley National Laboratory, Berkeley, California CA 94720, United States

**Keywords:** thermodynamics, templated mineralization, peptoid, calcium carbonate, synthetic polymer substrates, biomimicry, nanocomposites, scaling

## Abstract

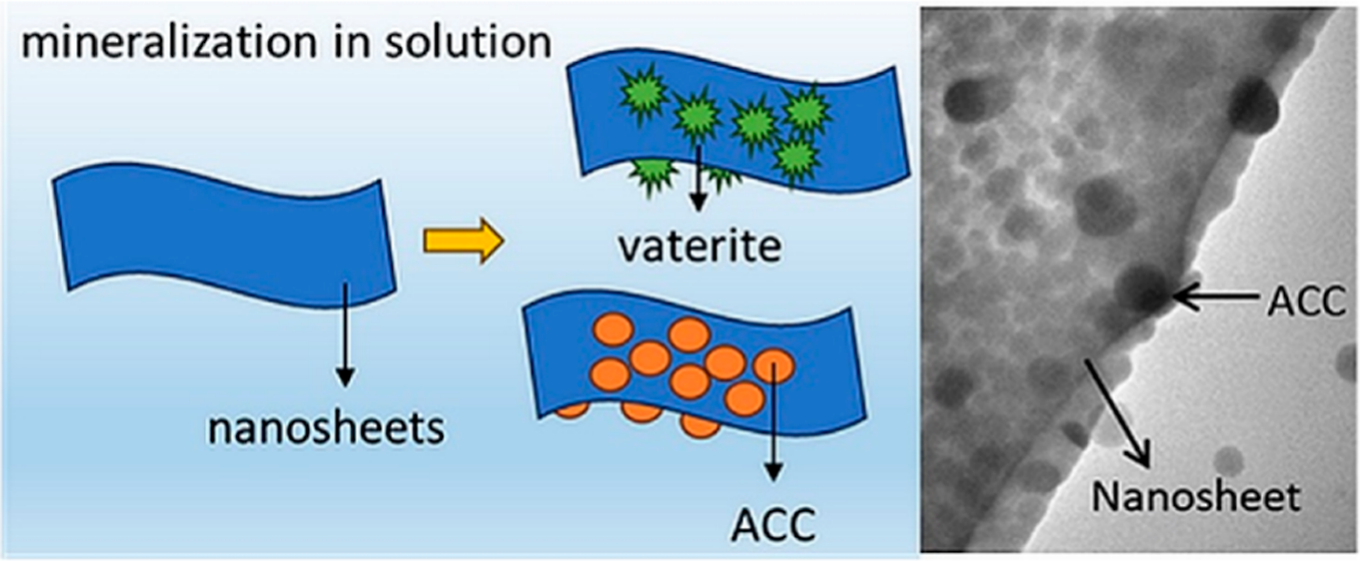

Mineral-polymer composites found in nature exhibit exceptional structural properties essential to their function, and transferring these attributes to the synthetic design of functional materials holds promise across various sectors. Biomimetic fabrication of nanocomposites introduces new pathways for advanced material design and explores biomineralization strategies. This study presents a novel approach for producing single platelet nanocomposites composed of CaCO_3_ and biomimetic peptoid (N-substituted glycines) polymers, akin to the bricks found in the brick-and-mortar structure of nacre, the inner layer of certain mollusc shells. The significant aspect of the proposed strategy is the use of organic peptoid nanosheets as the scaffolds for brick formation, along with their controlled mineralization in solution. Here, we employ the B28 peptoid nanosheet as a scaffold, which readily forms free-floating zwitterionic bilayers in aqueous solution. The peptoid nanosheets were mineralized under consistent initial conditions (σ_calcite_ = 1.2, pH 9.00), with variations in mixing conditions and supersaturation profiles over time aimed at controlling the final product. Nanosheets were mineralized in both feedback control experiments, where supersaturation was continuously replenished by titrant addition and in batch experiments without a feedback loop. Complete coverage of the nanosheet surface by amorphous calcium carbonate was achieved under specific conditions with feedback control mineralization, whereas vaterite was the primary CaCO_3_ phase observed after batch experiments. Thermodynamic calculations suggest that time-dependent supersaturation profiles as well as the spatial distribution of supersaturation are effective controls for tuning the mineralization extent and product. We anticipate that the control strategies outlined in this work can serve as a foundation for the advanced and scalable fabrication of nanocomposites as building blocks for nacre-mimetic and functional materials.

## Introduction

Biocomposites inspire bottom-up development of novel materials because they exhibit superior mechanical properties compared to the individual constituents.^1−4^ One biocomposite that has been heavily investigated is nacre, which is an internal layer of some mollusk shells that increases shell toughness and, thereby, protects the species. The toughness originates from the hierarchically ordered layered structure, which on the micrometer scale resembles a brick-and-mortar architecture. It is composed of 95 vol % of aragonite, a crystalline polymorph of calcium carbonate (CaCO_3_), and 5 vol % of organic material.^2^ The brick and mortar structure has been replicated using a variety of inorganic minerals (e.g., CaCO_3_ and clay) and organic polymers using a “layer by layer” assembly strategy^5,6^ or other surface-dependent strategies for material fabrication.^7^ However, surface-dependent fabrication is difficult to scale up. In this study, we apply different mineralization scenarios to form different CaCO_3_ phases on free-floating organic peptoid nanosheets. This is a new approach where the scaffold for tablet formation is organic, whereas others have used inorganic tablets and combined them with polymers.^8^ The ability to create nacre-mimetics is synthetically challenging, but mineralization of free-floating nanosheets in solution allows the production of a new (metastable) nanoscale intermediate. Our process offers an alternative method for homogeneous and stable mineralization of peptoid nanosheets in solution, which can result in a cheap, scalable, and controllable process for material manufacturing.

Peptoid polymers are *N*-substituted glycines that can be synthesized with sequence-specific control. This has been exploited to obtain specific configurations, one of them being ultrathin water-soluble bilayers known as nanosheets.^9−13^ Nanosheets are formed from linear peptoids that alternate between charged hydrophilic and hydrophobic functional groups. When the nanosheet is folded, the hydrophobic groups are hidden from the solution as an internal layer, while all hydrophilic groups are exposed on the sheet surface. Electrostatic interactions between the positively charged and negatively charged hydrophilic groups stabilize the sheet structure.^9^ One of the polymers known to form nanosheets is the B28 peptoid polymer, which is composed of 28 monomers with the hydrophilic groups organized in a block structure of seven carboxylic acid [*N*-(2-carboxyethyl)glycine] and seven primary amines [*N*-(2-aminoethyl)glycine] with 14 hydrophobic groups [*N*-(2-phenylethyl)glycine] alternating in between.^9,10^ The B28 nanosheets were first investigated for the fabrication of composite ceramics by Jun et al., who introduced templated CaCO_3_ growth on immobilized nanosheets by using the ammonia diffusion method.^14^ While the mentioned study set a precedent for nanosheet mineralization resulting in unique planar nanocomposites, it did not introduce a scalable production method or provide control over mineral product formation.

CaCO_3_ has three anhydrous polymorphs: calcite, aragonite, and vaterite, and a hydrous amorphous calcium carbonate (ACC) phase, which is a transient phase in aqueous systems.^15−20^ In solution, classical crystallization theory describes the formation of CaCO_3_ through a first-order phase transformation mechanism, where the solid phase forms to lower the chemical potential of the system. Here, the thermodynamic stability of the CaCO_3_ phases follows the order: calcite > aragonite > vaterite with ACC being the most unstable.^21,22^ During spontaneous precipitation at saturation above that of ACC, ACC is often the first phase to form. At room temperature, the spherical nm scale ACC transforms to μm-sized polycrystalline vaterite after seconds to minutes, during a dissolution–recrystallization process.^23^ In additive-free and well-stirred systems, vaterite transforms to thermodynamically stable calcite with a rhombohedral morphology in a matter of hours.

The different CaCO_3_ polymorphs exhibit distinct thermodynamic and kinetic stability regions. Despite this, their solubilities are relatively similar. Consequently, the presence of ions or organic compounds can inhibit the formation of a particular phase, leading to a wide array of variations within the CaCO_3_ system.^24−28^ The CaCO_3_ precipitation systems are sensitive to saturation conditions, spatiotemporal variations in supersaturation profile, and temperature. Previous work has demonstrated the regulatory roles of parameters such as the addition rate and sequence of precursor solutions, mixing efficiency, and reaction volume on both the crystallization pathway and resultant product.^29,30^ These factors affect the supersaturation profile and the nucleation and growth kinetics of the subsequent phases. Seeding has proven effective for polymorphic control and sustained growth of the seeds, hence bypassing the necessity for a substantial initial driving force to induce nucleation.^31^ Another strategy to obtain CaCO_3_ at a low driving force involves introducing a surface that decreases the interfacial free energy and the activation energy barrier for nucleation.^32,33^

Regardless of the supersaturation level aimed for, feedback-controlled systems can provide near-constant supersaturation levels throughout an experiment, thereby providing steady-state conditions for study of complex precipitation systems.^34,35^ For calcium carbonate, feedback-controlled systems have been used to investigate growth mechanisms and kinetics in seeded experiments.^35^ In contrast, batch systems operate with continuously depleting supersaturation profiles during precipitation, potentially leading to the formation of different phases as they traverse thermodynamic and kinetic stability regions of multiple phases.

In this study, we aimed to utilize different saturation states and spatiotemporal profiles during particle nucleation and growth to obtain different polymorphs and manipulate the extent of mineralization on peptoid nanosheets. We used thermodynamic speciation to identify conditions leading to vaterite formation in a batch precipitation system, and two feedback-controlled setups were utilized to promote ACC formation. In the model, solution speciation was calculated by considering mixing ratios of calcium and carbonate sources. Vaterite formation was obtained in a batch setup, where nanosheets were mineralized under depleting supersaturation, which are known to lead to the elimination of metastable phases and produce crystalline polymorphs.^36^ For the ACC inducing and bypassing systems, feedback-controlled setups ensured well-defined and near-constant supersaturation levels in the mixed liquid. We used scanning electron microscopy (SEM) coupled with electron-dispersive X-ray spectroscopy (EDXS) to simultaneously assess the morphology and elemental composition of the precipitates. We examined the precipitating calcium carbonate phases using X-ray diffraction (XRD) and transmission electron microscopy (TEM) combined with selected area electron diffraction (SAED). The speciation calculation code PHREEQC was used to understand the supersaturation development under different mineralization conditions and the resulting variations on the mineral content.

## Experimental Methods

### Peptoid Nanosheets

We used the solid phase submonomer method to synthesize B28 peptoid polymers using a commercially available Aapptec Apex 396 robotic synthesizer. We purified the polymers by reversed-phase HPLC and lyophilized the product. To obtain a 2 mM peptoid stock solution, we dissolved the lyophilized peptoids in a 2:1% v/v mixture of dimethyl sulfoxide (DMSO) and demineralized water. Peptoid nanosheets formed in an aqueous solution. To a 4 mL glass vial, we added 445 μL of deionized water (Milli-Q, resistivity >18.2 MΩcm), 50 μL of 100 μM aqueous solution of *tri*(hydroxymethyl)aminomethane (TRIS, Sigma-Aldrich) to buffer the solution to pH 8.0, and 5 μL 2 mM peptoid stock solution, resulting in a peptoid polymer concentration of 20 μM. To optimize the formation of peptoid nanosheets, we placed the vials in a simple rocker robot built after the principles presented by Sanii et al. and left the peptoid solution to form nanosheets for 72 h.^11^ After bilayer formation, we checked that the nanosheet formation had been successful by placing a 2 μL droplet of nanosheet solution on a freshly made gel of agarose (Sigma-Aldrich) and imaging the sample with an optical microscope (ZEISS Axio Imager). We operated the optical microscope with 10×, 20×, and 50× magnification objectives and in bright field reflecting light mode where white light is reflected from the sample surface and detected. The zeta potential of the nanosheets was measured in buffer solutions ranging from pH 6–10 (Table S1 and Figure S1).

Prior to the mineralization experiments, we dialyzed the peptoid nanosheets using a dialysis kit from Spectra/Por (Float-A-Lyser G2, MWCO 100 kDa, 1 or 5 mL), a process that removes free peptoid polymers and components left from the preparation process (e.g., salts, DMSO, and TRIS). We equilibrated the dialysis filters for 30 min in deionized water before adding peptoid nanosheet solution into the filters. During the first dialysis circle, we dialyzed the nanosheets in 0.1 mM aqueous TRIS solution at pH 8.0 (100× dilution from the peptoid nanosheet solution) to minimize drastic drops in ionic strength. For the following circles, we used deionized water, renewing the solution a minimum of three times. After 24 h, we terminated the dialysis process and used the nanosheets for experiments.

### Nanosheet Mineralization

We used deionized water to prepare CaCl_2_·2H_2_O (99%, Sigma-Aldrich), NaHCO_3_ (Sigma-Aldrich, > 99.7%), and Na_2_CO_3_ (Merck, 99.9%) solutions. We prepared a CaCl_2_·2H_2_O stock solution whose actual concentration was checked by using flame atomic absorption spectroscopy (PerkinElmer AAnalyst 800). Carbonate solutions were prepared freshly immediately before the experiments to ensure that pH did not change because of interactions with CO_2_ in air.

**Figure 1:**
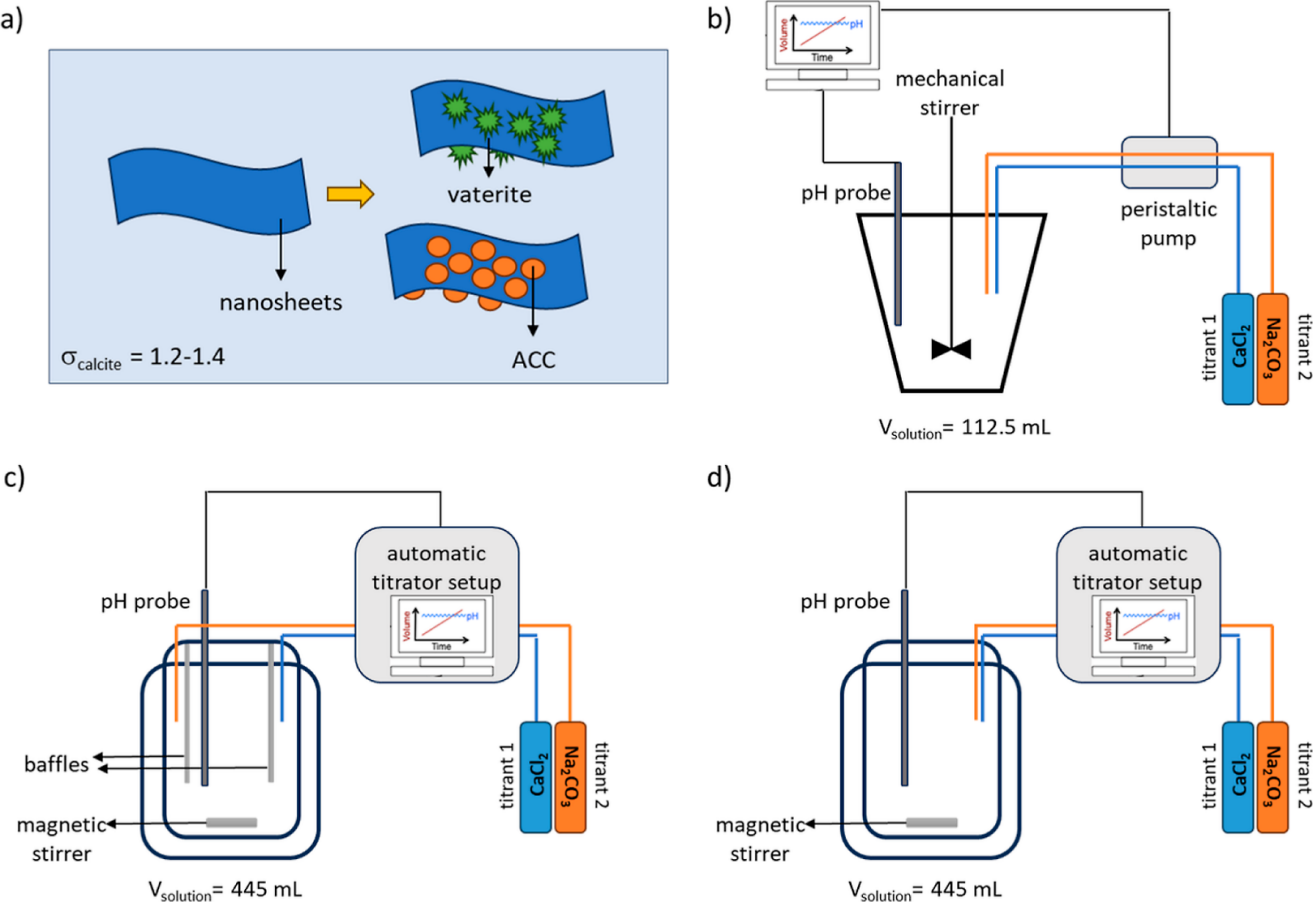
(a) Nanocomposites are formed by using organic peptoid nanosheets as scaffolds and mineralizing them either by ACC or vaterite via different experimental procedures. Schematics of different feedback control experiments are illustrated; (b) the standard feedback control setup, the modified feedback control setup operated at (c) sufficient mixing conditions, and (d) insufficient mixing conditions.

Different experimental setups were used to produce single platelet nanocomposites by mineralization of dispersed nanosheets (Figure 1a) and to investigate the effects of titrant mixing and subsequent fluctuations in the spatial distribution of supersaturation on mineralization reactions. Batch experiments that allowed for consumption of ions and a decrease in supersaturation during mineralization were employed in addition to two feedback control setups that continuously replenished supersaturation via titrant addition, with differences in titrant mixing procedure and probe sensitivity. In feedback control experiments, the standard setup was used for the verification of sheet mineralization and the modified setup allowed for variations in titrant mixing procedures (Figure 1b–d).

### Thermodynamic Calculations of Solution Speciation in Mineralization Experiments

We used the geochemical speciation code PHREEQC (28) with the phreeqc.dat database to calculate the speciation and define solution conditions to be used in experiments that would result in specific calcite supersaturation. In the input file, we fixed pH to 9.0, the calcium concentration to 1 mM, and the supersaturation (σ) with respect to calcite range between 1.2 and 1.4, with σ being defined
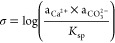
1

Here, a_*x*_ represents the activity of ion *x* and *K*_sp_, the solubility product for calcite, which by Plummer and Busenberg were reported to be 10^–8.48^ at 25 °C.^21^ From the output file, we obtain the total carbonate concentration needed to obtain the specified supersaturation and the ratio between NaHCO_3_ and Na_2_CO_3_ that result in pH 9.0 (Table S2). In addition, we used PHREEQC to determine the supersaturation state at 25 °C for vaterite (*K*_sp_ = 10^–7.91^) and for ACC (*K*_sp_ = 10^–6.40^).^21,22^ The value for ACC was derived using the aqueous speciation scheme in Plummer and Busenberg.^21^ Thus, the values of the solubilities are consistent.

Lastly, higher CaCO_3_ supersaturation levels may develop locally as solutions are mixed (initially in the UV–vis experiments and near the titrant addition tubes in the feedback control experiments). To probe the extent of such excursions in saturation, the supersaturation was calculated with PHREEQC as a function of solution mixture ratios for the initial mixing in the batch experiment and the mixing at the titrant addition tubes in the feedback control experiments.

### Batch Experiments

In the batch experiments, mineralization was monitored via changes in turbidity and measured with UV–vis spectroscopy. An Avantes AvaLight-DH-S-BAL light source, an Ocean Optics STS-UV spectrometer, and the software Ocean View were used to follow the attenuation of light as a function of mineralization. The reaction volume was 4.0 mL added to 4.5 mL cuvettes. To minimize the headspace, all cuvettes were sealed with parafilm to limit the interaction between solution and CO_2_ in air. For all experiments, we used an initial CaCl_2_ concentration of 1 mM, whereas the total carbonate concentration varied between 2.3, 3.0, and 3.6 mM prepared with a Na_2_CO_3_ to NaHCO_3_ ratio of approximately 1:9 (Table S2). Experiments were conducted for all three solutions, both with and without nanosheets. When dialyzed nanosheets were included, we used nanosheet solutions equivalent to a peptoid polymer concentration of 2.0 μM. During the UV–vis experiments, we used a constant wavelength of λ = 450 nm chosen because there is minimal interferences with water at this wavelength and the solution was mixed using a magnetic stir bar.^37,38^ Prior to an experiment, we obtained two reference spectra; (i) a background measurement, detecting the light coming from the setup itself, and (ii) a blank measurement of the carbonate solution (±nanosheets) where no reaction had occurred. These reference spectra were subtracted from the data as a part of the software data analysis. At the end of the nanosheet experiments, we prepared samples for SEM by placing a 2 μL droplet on a freshly cleaned Si-wafer purchased from TED PELLA (5 × 7 mm chips) and leaving them to dry.

### Feedback Control Experiments—Standard Setup

We mineralized peptoid nanosheets using a feedback control setup, inspired by the constant composition method developed by Nancollas.^34,39^ We used a cone-shaped glass reaction vessel with an overhead propeller (Metrohm 802) stirring at approximately 300 rpm. A pH electrode was inserted in the reactor and an inlet was used for titrants’ tubing (Figures 1b and S2). Before use, the glass vessel and propeller were cleaned with 0.5 M HCl followed by thorough washing in deionized water and drying using N_2_ gas. For experiments including peptoids, additional washing in ethanol and deionized water was added before drying. We calibrated the pH electrode using standard buffers of pH 4, 7, and 9 from Metrohm.

The total solution volume in the vessel was 112.5 mL, containing 1.0 mM CaCl_2_ and 2.0 mM total carbonate in a Na_2_CO_3_ to NaHCO_3_ ratio of approximately 1:9 (Table S2). For experiments with nanosheets, the carbonate solution included the dialyzed nanosheet solution. The total peptoid polymer concentration in the vessel was 2.0 μM. We initialized the experiment by adding CaCl_2_ as the last component. The addition induces a pH decrease followed by stabilization within ∼2 min at pH 9.01 ± 0.01. After pH stabilization, the automatic addition of CaCl_2_ and Na_2_CO_3_ titrants was initiated to buffer the pH decrease as a result of mineralization. During an experiment, calcium carbonate precipitates by the reaction
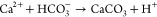
2thus changing the calcium and carbonate concentration in the solution and decreasing pH. The pH decrease is detected by the pH meter with a sensitivity of 0.01 units and triggers the injection of equal volumes of 100 mM CaCl_2_ and 100 mM Na_2_CO_3_ titrants to maintain constant pH and supply precursor ions, thus replenishing the solution supersaturation. Each experiment ran for a minimum of 5 h.

Samples were retrieved every hour by withdrawing ∼0.5 mL of the liquid using a syringe (inject) through an N20 butyl-rubber stopper cap, which had been installed on top of the setup to maintain a closed system. We placed 2 μL of the sample on a freshly cleaned Si-wafer purchased from TED PELLA (5 × 7 mm chips) for AFM and SEM investigations and a similar volume on a carbon-coated cobber grid (electron microscopy sciences, FCF200-CU, Formvar/Carbon 200 Mesh Copper) for TEM analysis. Note that it was not possible to freeze-dry nor vacuum filter the sheets due to their brittle nature and the samples were instead air-dried after deposition on a silicon wafer. The mineralization process was investigated also without the nanosheets in the same setup. At the end of experiments without nanosheets, the solution was vacuum filtered (filter pore size 200 nm), and no particles could be detected on the filter.

### Feedback Control Experiments—Modified Setup

All experiments were carried out in a magnetically stirred 0.5 L double-walled glass reactor with a flat bottom (Figures 1c,d and S3). The temperature was controlled by a water bath at 25 °C. The solution composition in the reactor was kept at a supersaturation level of σ_calcite_ = 1.2 as given in Table S2, where the total reaction volume was increased by four times. Two different mixing procedures denoted as insufficient and sufficient mixing was employed together with two levels of titrant concentrations (100 and 200 mM). Under insufficient mixing conditions, imitating the feedback-controlled standard setup, the tubings of the two titrant solutions were introduced from the same inlet under stirring at 200 rpm, and no baffles were present. Under sufficient mixing conditions, the titrant solutions were injected into the reactor from opposite sides under magnetic stirring at 300 rpm, and two baffles were attached to the lid to promote homogeneous mixing. Titrant addition was automatically prompted upon changes in solution pH by 0.002 units and was monitored together with solution pH via a Metrohm Titrando 902 setup. Solution supersaturation was confirmed and monitored by calcium and alkalinity analyses via offline titration at arbitrary time points, which showed less than a 10% deviation in concentration from the initial values during experiments. Samples were taken from the reaction medium at arbitrary time points for SEM imaging via placing drops of solution on silicon wafers and carefully blotting the excess liquid. Calcium ion and alkalinity concentrations in the vessel were monitored by titration (Mettler Toledo DL 53) of filtered solution samples with 10 mM EDTA and HCl, respectively. In the modified setup, the titrant addition curves for both mixing procedures reach significantly higher volumes compared to the standard setup because of the higher sensitivity of the pH probe and larger reaction volumes.

Control experiments were made for both feedback-controlled mineralization setups, where nanosheets or seeds were omitted, to investigate mineralization process in blank and effects of sampling process on experimental observations (detailed information is given in Supporting Information, Figures S4–S6).

### Characterization of Mineralized Nanosheets

SEM was employed to image drop-casted samples of mineralized nanosheets in all mineralization experiments, enabling monitoring of the extent of mineralization and the observation of morphological features of the precipitates. A coating layer to minimize the charge was not necessary. A Quanta 3D FEG SEM instrument with xT microscope control software was utilized, operating at an acceleration voltage of 5 kV and with an aperture of 30 μm. EDXS and TEM with ED was utilized to further characterize the mineralized nanosheets obtained from standard feedback control experiments. EDXS analysis was performed to enable chemical analysis, with the acceleration voltage increased to 10 kV. Data analysis was conducted by using the Aztec Software package. TEM imaging was conducted by using a CM20 Philips microscope operated at 200 kV, with electron detection facilitated by a Veleta CCD camera. In the modified feedback control setup, precipitation was observed in solution, and the reaction content was subsequently filtered at the end of the experiments. Hence, powder XRD analyses were carried out using a Bruker D8 Advance diffractometer in the 2θ range of 4–75°, with a step size of 0.013° and a step time of 0.67 s. The mineralization rates were monitored using well-established methodologies: optical density (OD) measurements for batch mineralization^40^ and titration curves in feedback control setups.^39^ An overview of experimental conditions and associated characterization methods is given in Table 1.

**Table 1:** Overview of the Nanosheet Mineralization Experimental Conditions, Characterization Methods, and Resulting Precipitates

mineralization setup	σ_calcite_	reaction volume	titrant concentration	precipitate on nanosheets	precipitate in solution	solid phase characterization	mineralization rate measurements
batch	1.2–1.4	4 mL		vaterite	vaterite	SEM	optical density measurements
feedback control-standard	1.2	112.5 mL	100 mM	ACC		SEM/TEM-ED/EDXS	titration rate
feedback control- modified	1.2	445 mL	100 mM	ACC-resembling, small spherical particles	calcite & vaterite	SEM/XRD	titration rate

## Results and Discussion

### Mineralization Pathways Following Different Mixing and Supersaturation Profiles in the Feedback Control and the Batch Systems

**Figure 2:**
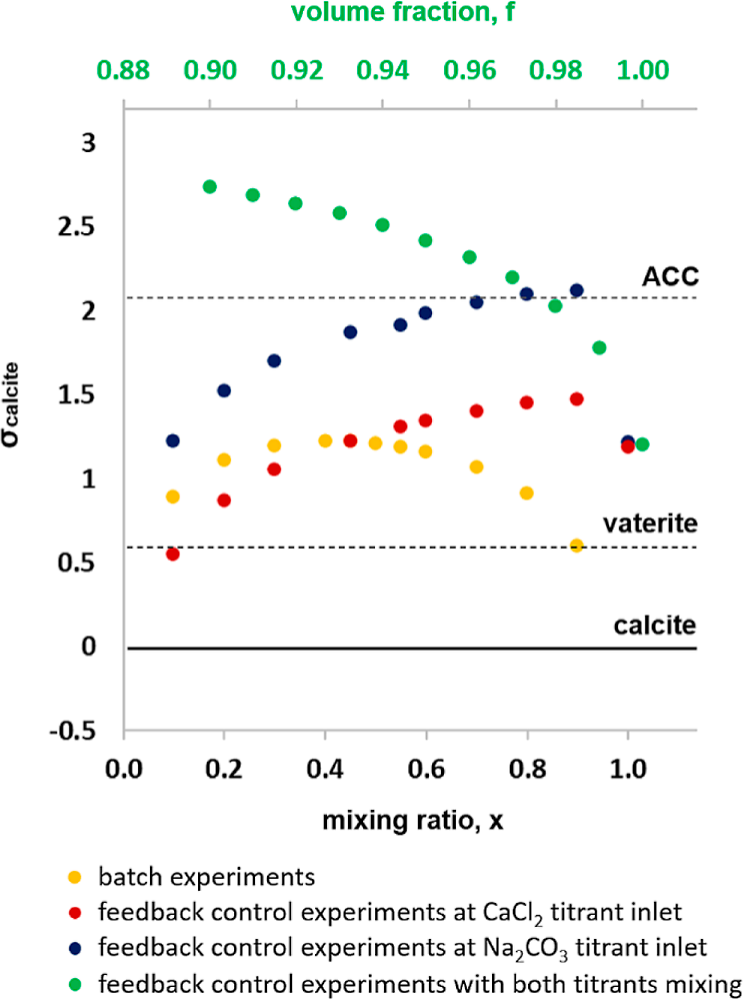
σ_calcite_ as a function of mixing ratio, *x*, calculated for σ_calcite_ = 1.2 performed in batch and feedback control experiments. For the batch experiments, *x* = 0.5 describes the nominal conditions of our experiment. For the feedback control experiments, *x* = 0.1 simulates mixing of individual titrant solutions with the reaction solution in a 1:9 volume ratio, whereas *x* = 1 simulates the reaction solution. For considerations of simultaneous mixing of both titrants, *f* = 0.90 denotes mixing of reaction solution with both titrants in a 9:1 ratio. Black horizontal lines mark the solubility limits of the calcium carbonate phases.

To induce CaCO_3_ mineralization on the sheet surfaces, it is important to have a low supersaturation, where the surface would provide an effective decrease in interfacial energy and potentially drive precipitation toward the sheet surfaces rather than nucleation in solution. A homogeneously mixed solution with the target σ_calcite_ of 1.2 is supersaturated with respect to vaterite, but undersaturated with respect to ACC (σ_ACC_ = −0.88). However, undersaturated phases may form as a result of the decrease in interfacial free energy some favorable surfaces can induce.^24^ In cases when a solution is poorly mixed, sporadic higher local supersaturation levels can occur and also induce the formation of phases that are undersaturated in well-mixed bulk conditions. We used chemical speciation calculations (PHREEQC) and consideration of mixing to explore the possible drivers of different mineralization products forming on nanosheets in the feedback-controlled setups versus the batch experiments. The influence of a surface was not considered in the model. Figure 2 shows a plot of σ_calcite_ as a function of solution mixing ratio, *x*, expressed as (i) the volume fractions of calcium and carbonate solutions in the batch experiments and (ii) the volume fraction of added CaCl_2_ and/or Na_2_CO_3_ titrant solutions to the reaction solution in the feedback control experiments. For the batch experiment (Figure 2, yellow dots), *x* = 0.5 is the fully mixed solution, whereas *x* = 0.9 represents a CaCl_2_ rich portion during mixing and *x* = 0.1 a carbonate-rich portion. For speciation calculations in feedback control experiments, *x* = 0.1 is close to the inlet of the CaCl_2_ (Figure 2, red dots) and Na_2_CO_3_ (Figure 2, blue dots) titrants, and *x* = 1 represents the reaction solution. In a second scenario for feedback control experiments (Figure 2, green dots), we assumed the simultaneous mixing of both titrant solutions with the reaction solution in small volume fractions, *f*, from 0 to 0.1, where *f* = 1 represents the reaction solution.

For the batch experiments, Figure 2 (yellow dots) shows that σ_ACC_ is not exceeded during mixing, meaning that ACC formation in the solution is predicted to be thermodynamically impossible at any mixing ratios. In addition, through mineral formation, supersaturation, and pH in the reaction medium will constantly be depleting from the initial values. Under the initial condition, the solution is supersaturated with respect to vaterite (σ_vaterite_ = 0.62), which is also the kinetically most favorable polymorph of CaCO_3_ at the reaction temperature.^41^ In contrast, calculated σ_ACC_ is exceeded in the feedback control experiments at the Na_2_CO_3_ titrant inlet (blue dots) when the influent solution has mixed at a 1:5 to 1:10 ratio with the bulk solution. When simultaneous mixing of both titrants was considered (green dots), even very low volume fractions were shown to result in surpassing the solubility limit of ACC. Thus, when mixing is insufficient, ACC formation is predicted to be thermodynamically allowed for a period of the mixing process due to local high supersaturation zones. In contrast, well-mixed titrants are not expected to produce ACC as homogeneous solutions at σ_calcite_ = 1.2 are undersaturated with respect to ACC. Controlling mineralization at high supersaturation levels is difficult due to the inherent metastability of solution and initially nucleating phases.

Nanosheets were mineralized via different experimental procedures and variations in mineral content was observed in correlation with the thermodynamic calculations (Table 1). In the following, we probe how mixing can be used to induce local supersaturation and show that sheets are able to induce stabilization of otherwise transient ACC.

### Mineralization of Nanosheets in Batch Experiments with UV–Vis Measurements

**Figure 3:**
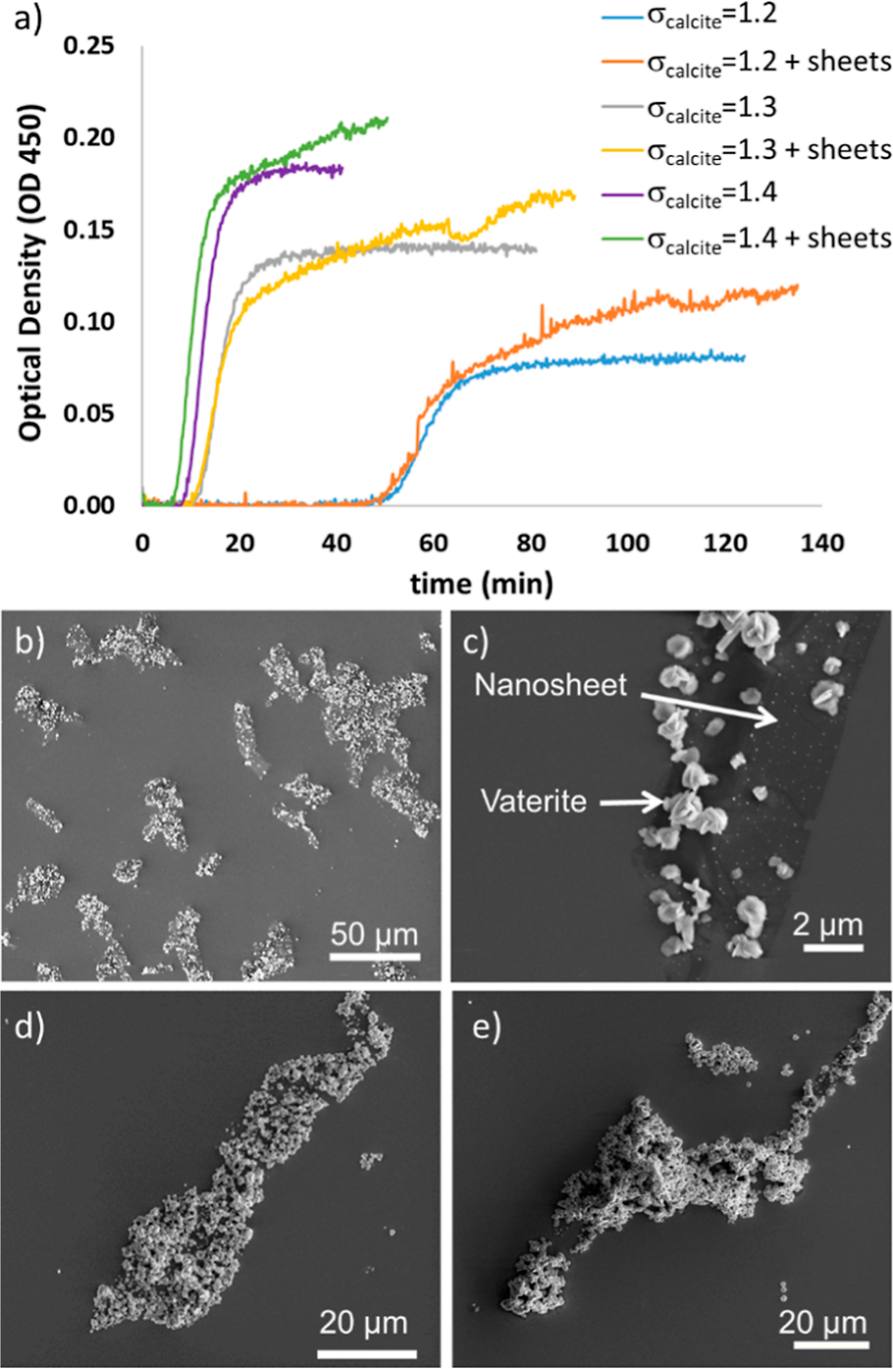
(a) OD measurements in solutions at λ = 450 nm as a function of time with and without nanosheets, SEM image of mineralized nanosheets after batch experiments at (b,c) σ_calcite_ = 1.2, (d) σ_calcite_ = 1.3, and (e) σ_calcite_ = 1.4. The large precipitates as marked in (c) show a typical vaterite morphology.

The turbidimetric scattering of light in a supersaturated solution varies as precipitation processes take place and we compared OD as a function of time for solutions prepared with and without nanosheets for specific σ_calcite_ levels (σ_calcite_ = 1.2, 1.3, and 1.4).^40,42,43^ With this approach, we get information on both the induction time for the formation of minerals, mineral growth rates, and any subsequent Oswald ripening process. In substrate-free systems, these processes are commonly observed to have an S-shaped curve.^37^ During the induction time, the curve is semihorizontal, and when the nucleation occurs and growth takes over, the curve becomes steep and flattens out when the system has equilibrated to a saturated solution with no further ripening of the precipitate. This S-shape is usually assigned to the precipitation of a single phase, whereas pronounced fluctuation in OD as a function of time results from phase transformation.^37,40,42,43^ Comparing the measured values of a pure system with a solution at the same σ_calcite_ level with nanosheets, the induction time and initial increase in OD values were very similar (Figure 3a). The similar shape of the scattering curves and the same precipitation product suggest that the nucleation behavior of CaCO_3_ was not greatly affected by the presence of nanosheets in the batch setup. However, after the point in time where the pure system reaches a plateau, clear differences in OD were apparent between solutions with and without nanosheets. In experiments with nanosheets, the OD values continued to increase after CaCO_3_ precipitation, which could be explained by a continuous accumulation of CaCO_3_ particles at the surface of the nanosheets or by the ripening of accumulated particles. Both scenarios would increase the “particle size”, e.g., size or volume of mineralized nanosheets, and thereby result in an increase in OD.

SEM images of the mineralized nanosheets from the batch experiments showed increased particle accumulation on their surface as a function of increasing σ_calcite_ (Figure 3b–e). At a σ_calcite_ of 1.2, which is the same saturation as used for the feedback control mineralization, the nanosheets in the batch experiments were not fully covered with particles and very little precipitation was observed outside the nanosheets (Figure 3b,c). At higher saturation indices, the sheets became more densely packed with the precipitates (Figure 3d,e). The morphology of the CaCO_3_ observed in the batch experiments typically resembled vaterite, and their sizes were larger than the ACC observed in the feedback control experiments.

Whether the vaterite particles nucleated on the sheets and transformed via a precursor phase that settled there or if the vaterite formed in solution and subsequently associated with the nanosheet is challenging to access. However, according to the thermodynamic modeling, no ACC would be stable to form in solution. It is unclear from the experiments, though, if the ACC could form on the sheet surface as a result of a reduced interfacial free energy. However, considering the resemblance of the UV–vis growth curves between systems with and without added sheets, we find it likely that vaterite formed in solution, subsequently associated with the sheets, and grew larger there. In addition, the morphology of the vaterite particles does indicate that the particles are flat on the side interacting with the sheet. Considering that vaterite has a spherulitic growth, a flat surface on a vaterite crystal strongly indicates that the particles grew there. Particles formed on the side of the sheet facing down during imaging induced fragmentation of the sheets during drying (Figure S7). During drying of the samples, the particles would have increased in size as a result of the increasing supersaturation. The batch experiments showed that the sheet presence did not influence the nucleation and growth behavior drastically, while different secondary processes were evoked.

### Mineralization of Nanosheets in Feedback-Controlled Standard Setup with Insufficient Mixing

**Figure 4:**
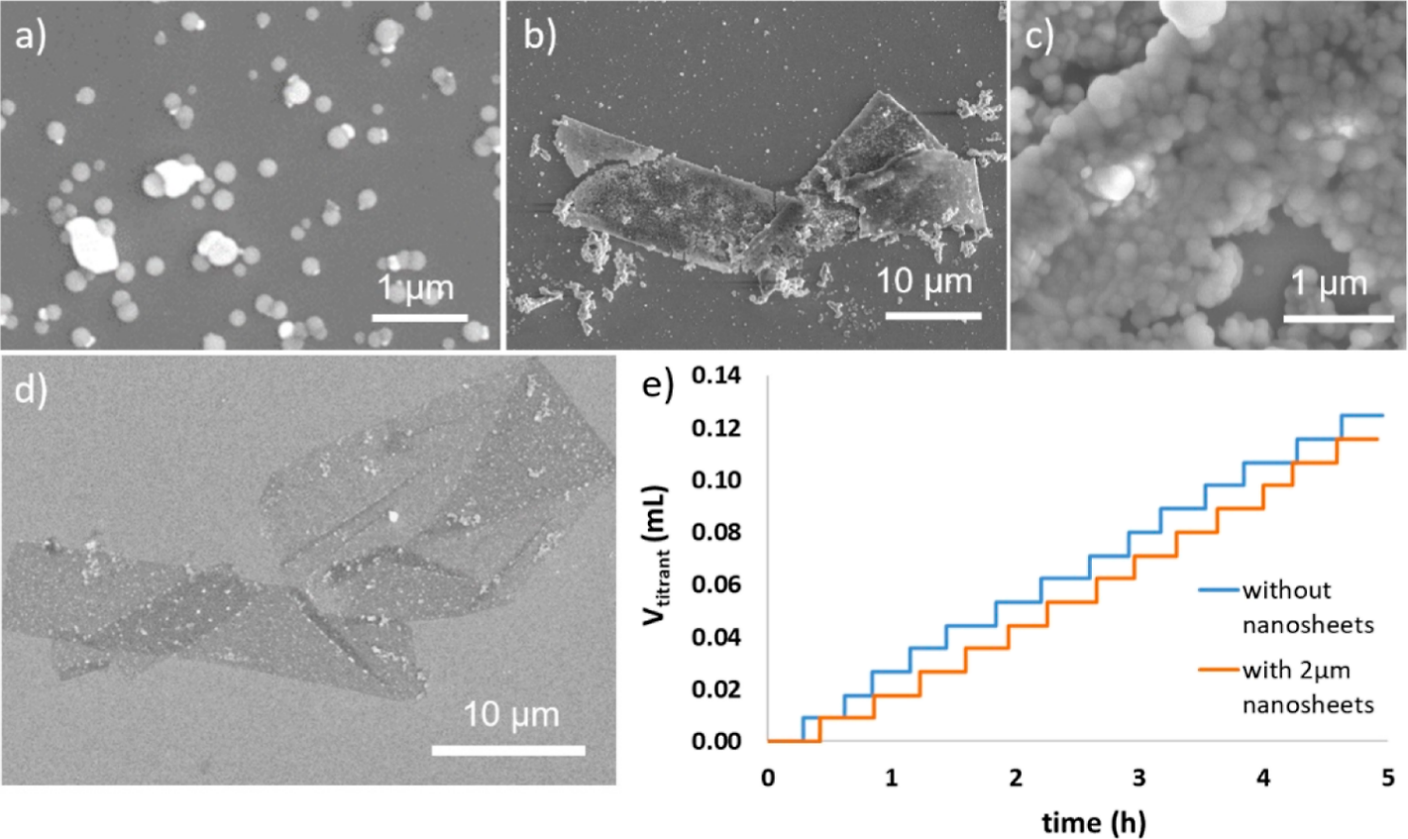
SEM images of mineralized peptoid nanosheets in the standard feedback control setup with σ_calcite_ = 1.2 and pH 9.0 and insufficient mixing, (a) sample taken at the end of experiments without nanosheets, (b,c) mineralized nanosheets collected at 4 h, (d) mineralized nanosheets collected at 2 h, and (e) titration curves of experiments with and without the nanosheets.

Using the feedback control experiments with insufficient mixing, we expected to induce ACC formation (Figure 2) near the inlets. Without nanosheets, no precipitation was observed when the solution was filtered through 200 nm pore size filter paper at the end of the experiments. When sampling was done via drop-casting, a small amount of precipitate was observed on wafers (Figures 4a and S4). With nanosheets added, the sampled nanosheets were heavily covered with spherical particles at a much larger coverage than was observed in the experiments without nanosheets (Figure 4b). The precipitated particles ranged from 50 to 400 nm in diameter (Figure 4c). The spheres appear to be present on both sides of the folded nanosheet, indicating that they mineralized in solution. Significantly more precipitate could be observed on the sheets after 4 h of mineralization compared to the coverage of particles on the sheets at 2 h (Figure 4d). The increased coverage over time indicate that the particles were accumulated over the course of the experiment duration. SEM images of sheets prior to the mineralization are shown in Figure S8. Because of the drop-casting approach used for sampling (to preserve the sheets), we conducted a series of experiments to study particle precipitation during drying (Figure S6). In short, we observed that nanosheets were not heavily mineralized upon drying, and increased supersaturation during drying induced large calcite or vaterite crystals in addition to a minor amount of the smaller spherical particles on the nanosheets. Thus, the observed overgrowth in the experiment did not result from drying.

During the experiments with or without nanosheets, the volume of titrants added was similar, despite the observable difference in the amount of precipitation (Figure 4e). This can be due to (i) limited mineralization on the sheets, (ii) mineralization of the glassware in the pure system, and (iii) possible CO_2_ intrusion into the system. Although the reaction medium is sealed and the air volume on top of the solution is minimized to limit this effect; in such long reaction times as 5 h, it is not possible to completely prevent CO_2_ absorption from the air, which would prompt pH decrease and consequent titrant addition.

**Figure 5:**
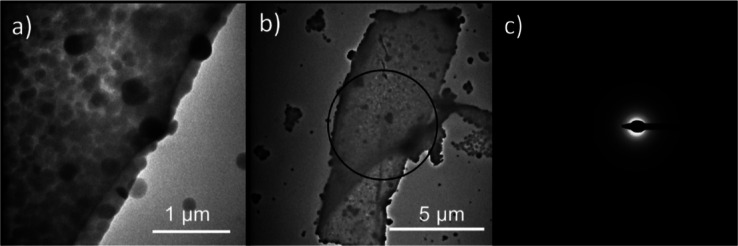
(a,b) TEM images of peptoid nanosheets mineralized in the standard feedback control setup using σ_calcite_ = 1.2 and pH 9. The circle in (b) indicates the area for the SAED. (c) SAED showed no diffraction spots, indicating an amorphous phase.

TEM images of the mineralized nanosheets showed the thickening of sheets caused by mineralization (Figure 5). SAED of the mineralized nanosheets showed diffuse rings, which is indicative of an amorphous phase (Figure 5c). EDXS confirmed that the amorphous particles contained Ca and C (Figure S9). The chemical composition, shape, size, and crystallinity (as inferred from the SAED) indicate that the particles were ACC. When nanosheet were aged in the reaction solution after mineralization for 10 days, ACC was recrystallized into vaterite as inferred from the particle morphologies (Figure S10).

In experiments without nanosheets a smaller number of precipitates resembling ACC in size and morphology is found on the wafers compared to amount of mineralization on nanosheets (Figure 4a). Since ACC is undersaturated at the bulk reaction conditions, we assume the precipitate represents drying artifacts due to drop-casting. They could form as a result of concentration gradients as a result of insufficient mixing, but such precipitation would dissolve again once mixed in with the rest of the solution (Figure 2). Considering the amount of the precipitate on the sheets building up over time, we expect that the ACC on the sheets formed in solution and was subsequently scavenged by the sheets, where they are stabilized.

### Role of Mixing and Titrant Concentration for the Mineralization Kinetics and Products

**Figure 6:**
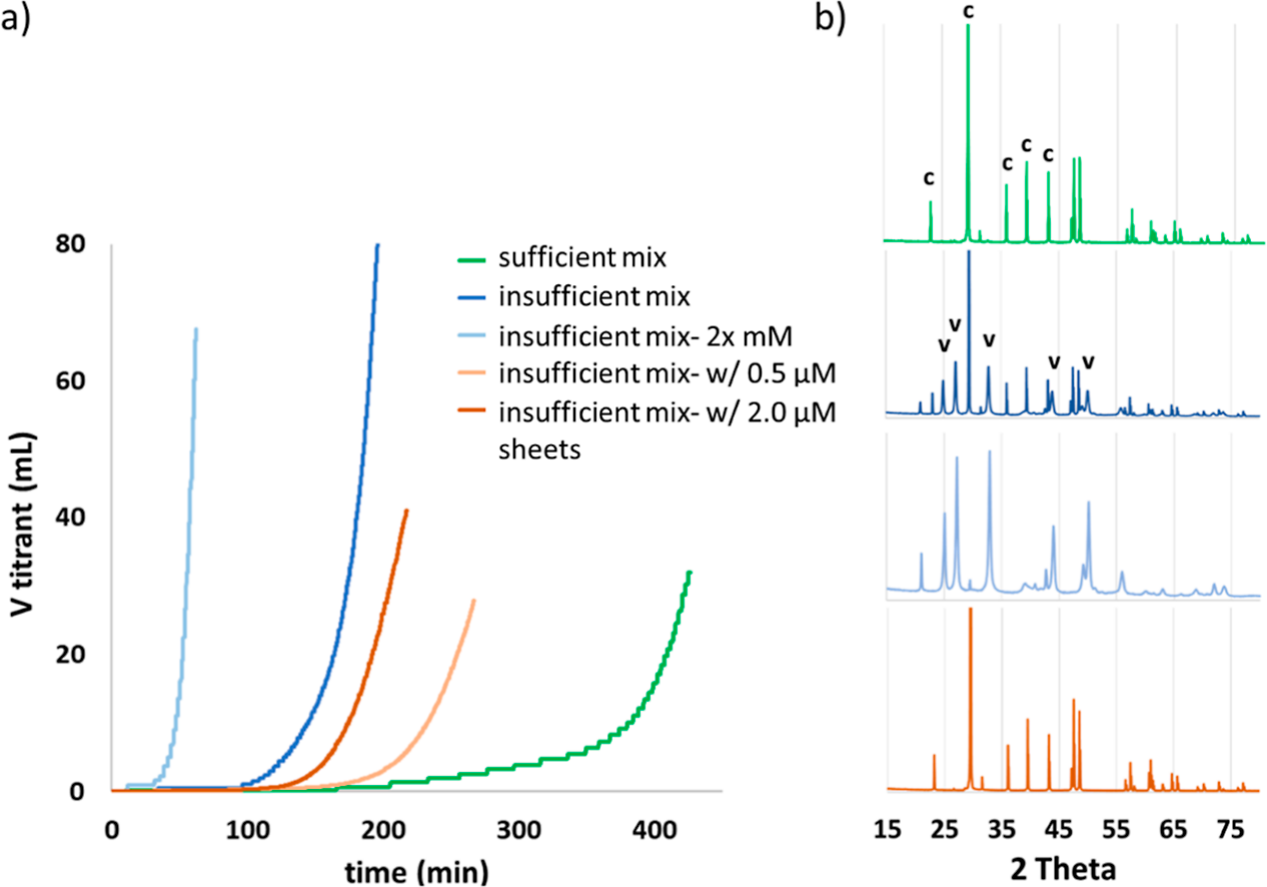
(a) Titrant addition curves as a function of time during modified feedback control experiments showing the rate of mineral formation. (b) XRD spectra of final precipitates from color-coded varying experiments. The significant peaks of calcite (c) and vaterite (v) polymorphs are marked on the spectra.

To further explore the controls on CaCO_3_ mineralization on the nanosheets, we used a modified feedback control setup. The modified setup allowed us to test the effects of mixing conditions at an increased pH probe sensitivity (0.002 versus 0.01 units) and a larger reaction volume, which resulted in the addition of more than 2 orders of magnitude higher volumes of titrants compared to the standard setup. Consequently, the differences in mineralization kinetics induced by the nanosheet presence were better observed. The modified setup was used to investigate the effects of mixing and titrant concentrations (100 and 200 mM) for the mineralization kinetics and products for systems with and without nanosheets (Figure 6). The experiments with sufficient mixing conditions and low concentration titrant and without nanosheets showed the longest induction time for detectable mineralization (150 min) as well as the lowest titrant addition rate of all the experiments (Figure 6 green curve). The modified setup allowed for precipitation in solution, which was not observed in the standard setup, probably as a result of higher numbers of nuclei formation with the increased reaction volume. SEM images and XRD analysis revealed that the precipitate was a mixture of calcite and vaterite crystals throughout the reaction (Figures 6b and S11).

At insufficient mixing conditions without nanosheets (rate and configuration similar to the standard setup), the induction time decreased and the mineral formation rate and vaterite content of the final precipitate increased (Figure 6, compare dark blue with the light blue color). The increasing mineral formation rate can be explained by the high local concentration at the proximity of titrant inlets caused by the insufficient mixing condition, which inherently would decrease the induction time and increase mineralization rate. According to the thermodynamic calculations (Figure 2) both vaterite, as the kinetically preferred phase at room temperature, and ACC were possible phases to appear in solution. Although ACC was not isolated at any point of SEM inspection, we cannot rule out its possible emergence in the system under high local supersaturation followed by dissolution or fast transformation to vaterite as the main phase ACC transforms to at pH 9 and 25 °C.^25,40^

Doubling the titrant concentration for an insufficient mixing experiment without nanosheets (light blue, Figure 6), decreased the induction time further (at 15 min), increased the mineralization rate and showed dominance of vaterite in the final precipitate (sampled at 60 min). SEM images of samples from the initial reaction point of ∼20 min showed precipitates with spherical morphologies and comparable size to ACC in addition to vaterite plates (Figure S12). The increased mineralization rate and faster induction time of the insufficient mixing system demonstrate that local supersaturation fluctuations can indeed accelerate mineral formation.

**Figure 7:**
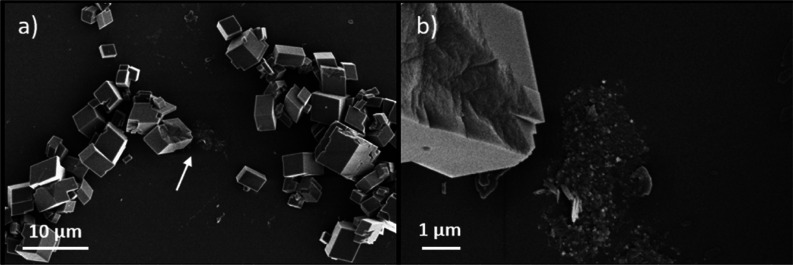
SEM images of precipitates from modified feedback control experiments with insufficient mixing in the presence of 0.5 μM nanosheets. Area marked by the arrow in (a) is shown at a higher magnification in (b) to show the different characteristics of precipitates in solution and on the nanosheets.

Under insufficient mixing conditions and with nanosheets, the titration curves showed slower mineralization rates and longer induction times compared to sheet-free experiments (orange vs blue lines in Figure 6). This trend was most pronounced at low sheet concentration (0.5 μM, yellow curve, Figure 6). SEM images and XRD data showed that the precipitate in the bulk solution was mainly calcite. In contrast, SEM images showed that the sheets were mineralized with spherical small particles (resembling ACC) (Figure 7). It is difficult to deduce a direct dependence of mineralization rate on sheet concentration since the titration rate is affected by multiple processes, such as precipitation of calcite and ACC, and possible phase transformations in solution. However, the difference in induction time and mineralization rate between the experiments with the different sheet concentrations shows that the sheets play a role in the mineralization. The increased induction time in the presence of sheets during insufficient mixing can be caused by scavenging and stabilization of ACC by the sheets, preventing recrystallization of the amorphous phase to vaterite and the associated accelerated consumption of supersaturation from the bulk solution. The mineralization product in solution changes to calcite compared to the dominance of vaterite in the sheet free system. The slower mineralization kinetics of calcite is reflected in the decreased rate and volume of the titrant addition.

The feedback-controlled system with insufficient mixing conditions and the presence of nanosheets induces ACC mineralization and stabilization. In the absence of the sheets, the unstable ACC either transforms to crystalline phases at a rate governed by the growth of the stable phase or dissolve. In contrast, in the presence of the nanosheets, ACC is stabilized, as reflected both by the decreased mineralization rates, and lower vaterite content in the bulk of the samples with nanosheets. Stabilization of the transient amorphous phase in solution via organic components is a common phenomenon, mainly via bulk incorporation or surface adsorption of additives.^44^ Biogenic minerals may occur as thermodynamically unstable amorphous forms that are templated and stabilized by the organic matrix, such as ACC found in crustacean exoskeletons.^45^ Widespread work in synthetic systems showed induced formation and prolonged lifetimes of ACC in the presence of organic molecules, and when precipitated on self-assembled monolayers, similar to our results.^46−48^ Additionally, in the solution we expect not only peptoid nanosheets but also singular peptoid polymers, which could stabilize the ACC similar to some amino acids and citrate by surface adsorption.^37,43^ Although the concentration of individual polymers is low compared to the concentrations of organic substances in these studies, Chen et al. have shown that a peptoid concentration <1 μM is sufficient for alteration of CaCO_3_ mineralization.^49^ Several studies have suggested multiple mechanisms on how organic molecules with charged functional groups provide stabilization for amorphous materials.^44,50−52^ Prolonged lifetimes of ACC can be a result of decreased heterogeneous nucleation rates of the crystalline phases on ACC, or hindered dissolution of the transient phase.^44^ A recent study associated the ACC stabilizing effect of carboxylated nanocellulose with the carboxyl content and rigid geometry, responsible for strong calcium binding and limiting the freedom of ACC, respectively.^53^ Similar mechanisms may be active in the stabilization of ACC via B28 nanosheets due to their carboxyl groups and structural features. Yet, such mechanistic insights are beyond the scope of this work, and further explanations cannot be offered within the experimental data of this study.

## Conclusions

This study presents a novel strategy for obtaining a biomimetic nanocomposite composed of peptoid nanosheets and CaCO_3_. Using a solution composition of σ_calcite_ = 1.2, batch experiments resulted in the mineralization of nanosheets with vaterite. Under same initial conditions, mineralization in feedback control experiments led to the complete coverage of the nanosheet surface with ACC under specific conditions of reaction volume, titrant addition, and mixing. The difference in the primary mineralizing phase for the two types of experiments is explained through chemical speciation calculations, which suggests that supersaturation fluctuations induced by insufficient solution mixing are responsible for the amorphous CaCO_3_ phases observed. The study also suggests a mineralization pathway where ACC is stabilized by the nanosheets. While the exact involvement of free peptoids or peptoid nanosheets in the stabilization of the ACC phase has still to be uncovered, it is an interesting perspective in light of the increasing amount of literature pointing toward growth pathways involving a precursor phase.

Our results demonstrate that nanocomposites with varying mineral composition can be prepared in solution by temporal and spatial modulations of supersaturation. Both the ACC- and vaterite-coated nanosheets offer potential versatile assembly units to be employed as metastable planar intermediates for the assembly of composite materials with a brick-and-mortar microstructure. Peptoid nanosheets have demonstrated their utility as robust, high-surface area scaffolds that can be tailored for specific applications, such as imparting antimicrobial properties and enabling molecular recognition.^54−56^ When mineralized with a transient amorphous phase, the mechanical properties of these nanosheets can be tuned as a function of the processing conditions. In nacre, ceramics used as bricks provide strength, and soft polymers used as mortar provide ductility and energy redistribution under stress. The remarkable mechanical properties stem from nacre’s hierarchical structure and organic–inorganic interface.^57,58^ The mineralized nanosheets could offer similar properties by enabling analogue mechanisms of energy dissipation, crack deflection, and plastic deformation when assembled into a hierarchical microstructure. By integrating these attributes, lightweight, durable, and functional nanocomposites can be realized, offering versatility for a wide range of applications. However, it is important to note that nacre’s exceptional toughness and tensile strength are also attributed to its layered assembly of high-aspect ratio and nanograined aragonite plates, a structural characteristic absent in nanosheets mineralized by ACC or vaterite.^59^ The experimental conditions leading to mineralization of ACC or vaterite on nanosheets, either directly via solution mineralization or by aging, are outlined and can be used for further development of synthetic procedures for scale up fabrication of nacre-mimetic materials. The mineralized nanosheets might be suitable for self-assembling into larger scale structures by filtration or gravity concentration methods, and present a potential for rational design of future high-performance materials.^60,61^

## Supporting Information Available

Zeta potential of nanosheets, PHREEQC calculations for mineralization conditions, schematics of feedback control experiments for the standard and modified setups, reference studies of CaCO_3_ precipitation to investigate drying artifacts, SEM images showing peptoid nanosheets and their fragmentation, EDXS of mineralized nanosheets in feedback-controlled standard setup, and SEM images of aged nanosheets mineralized in feedback-controlled standard setup (PDF)
