# Supplementary material for: Exploiting Saturation Regimes and Surface Effects to Tune Composite Design: Single Platelet Nanocomposites of Peptoid Nanosheets and CaCO3
Source: ACS Appl Mater Interfaces. 2024 Apr 3;16(15):19496–506. doi: 10.1021/acsami.4c00434 (PMC11040536; doi:10.1021/acsami.4c00434)
Supplement: Supplementary file 1 — am4c00434_si_001.pdf [file am4c00434_si_001.pdf]

Supporting Information

for

Exploiting Saturation Regimes and Surface Effects to Tune Composite Design: Single Platelet Nanocomposites of Peptoid Nanosheets and $CaCO_3$

*Seniz Ucar *[#a,b], Anne R. Nielsen [#c], Biljana Mojsoska [d], Knud Dideriksen [c, e], Jens-Petter Andreassen [a], Ronald N. Zuckermann [f], and Karina K.Sand* [c, g]*

[a] Department of Chemical Engineering, Norwegian University of Science and Technology, Trondheim, 7491, Norway

[b] Department of Metallurgical and Materials Engineering, Middle East Technical University, Ankara, 06800, Turkiye

[c] Nano-Science Center, Department of Chemistry, University of Copenhagen, Copenhagen, 2100, Denmark

[d] Department of Science and Environment, Roskilde University, Roskilde, 4000, Denmark

[e] Current address: Geological Survey of Denmark & Greenland (GEUS), Copenhagen K, 1350, Denmark

[f] Biological Nanostructures Facility, The Molecular Foundry, Lawrence Berkeley National Laboratory, Berkeley, California, CA 94720, USA

[g] Current address: GLOBE Institute, University of Copenhagen, Copenhagen, 1350, Denmark

[#] Joint first author position

* Corresponding author

*Karina K. Sand kks@sund.ku.dk

*Seniz Ucar senizu@metu.edu.tr

TABLE OF CONTENTS:

ζ-potential of nanosheets	3
PHREEQC calculations for mineralisation conditions	4
Feedback control experiments- standard setup	5
Feedback control experiments- modified setup	5
Reference study of $CaCO_3$ precipitation in standard feedback control setup	6
Reference study of $CaCO_3$ precipitation to investigate drying artifacts	6
Fragmentation of nanosheets	8
Peptoid nanosheets	8
Electron dispersive X-ray spectroscopy (EDXS) of mineralized nanosheets in standard feedback control setup	9
Aging of nanosheets mineralized in standard feedback control setup	9
Reference study of $CaCO_3$ precipitation in modified feedback control setup	10

ζ-potential of peptoid nanosheets

To measure the ζ-potential of nanosheets, we prepared nanosheets in buffers ranging from pH 6-10 (Table S1).[1] We made the nanosheets in the rocker as explained in the main text and sonicated the nanosheets for 15 min in a sonication bath to decrease the sheet size. We used deionised water to prepare stock solutions of the buffers in Table S1 with all compounds purchased from Sigma Aldrich. We calibrated the pH meter (Metroholm) using standard buffers of pH 4,7 and 9 and adjusted the pH of the buffers using HCl or NaOH. We dialysed the nanosheets overnight in 10 mM of each of the buffers using the dialysis kit from Spectra/Por® (Float-A-Lyser G2, MWCO 100 kD, 1 mL). We added 700 μL of the dialysed nanosheets to a disposable folded capillary cell and placed the cell in a Malvern Zetasizer Nano-ZS used to conduct the measurements. We equilibrated the sample at 20 °C for 300 s and conducted the electrophoretic mobility measurements with five repetitions per sample. After the experiment, we applied the Smoluchowski approximation to the data and obtained ζ-potential of the nanosheets (Fig. S1).

Table S1: Buffers used for ζ-potential measures in the pH interval of 6-10.

	Buffers
pH 6	MES (2-(*N*-morpholino)ethanesulfonic acid)
pH 7	MOPS (3-(*N*-morpholino)propanesulfonic acid)
pH 8	TRIS (tris(hydroxymethyl)aminomethane)
pH 9	AMPD (2-Amino-2-methyl-1,3-propandiol)
pH 10	CAPS (*N*-cyclohexyl-3-aminopropanesulfonic acid)

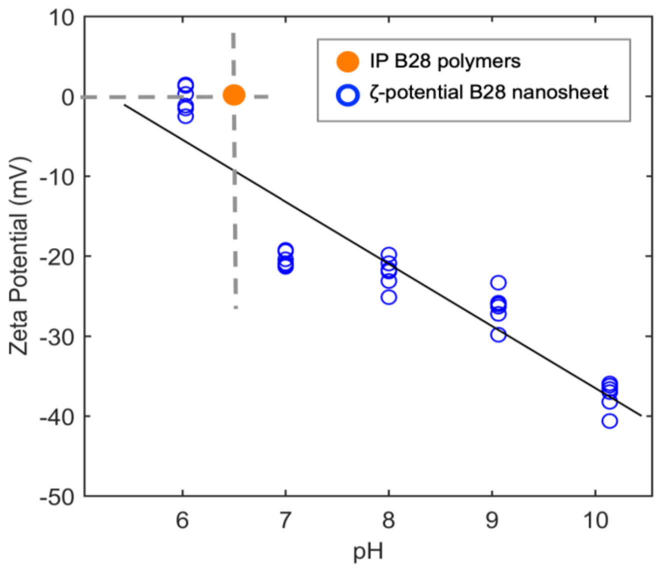

Figure S1: The isoelectric point (IP) of the nanosheets is below pH 6.5, which means that the nanosheets at mineralisation conditions (pH 9) are negatively charged. As reference, the IP of single B28 polymers is also plotted in the figure.[2]

PHREEQC calculations for mineralization conditions

Table S2 shows the thermodynamic calculations for solution conditions used for mineralization experiments. We calculated σ for the different calcium carbonate polymorphs using K_{sp} calcite = $10^{-8.48}$ and K_{sp} vaterite = $10^{-7.91}$, and K_{sp} ACC = $10^{-6.40}$.[3, 4] The calculated ion activities are given by $\{Ca^{2+}\}$ and $\{CO_3^{2-}\}$. We ran all calculations without equilibrating with CO_2 in air (Keeling curve 2018, 410 ppm, $\log(P_{CO2})$ = -3.39), which means the listed $\log(P_{CO2})$ in the table show that all solutions are undersaturated with respect to CO_2. To minimise the effect of this, we sealed all experiments using parafilm and filled containers used during mineralization as much as possible during experiments. Lastly, Max precip shows the maximum precipitation given in mol/L when solutions are set to change form supersaturated ($\sigma_{calcite}$ = 1.2-1.4) to calcite saturated ($\sigma_{calcite}$ = 0.0), which gives some idea to maximum material fabricated.

Table S2: PHREEQC of the final mixed solutions.

	$\sigma_{calcite}$	$\sigma_{vaterite}$	σ_{ACC}	$\{Ca^{2+}\}$	$\{CO_3^{2-}\}$	$Log(P_{CO2})$	Max precip (mol/L)
Solution 1	1.20	0.62	-0.88	6.66E-04	7.88E-05	-3.96	2.360E-04
Solution 2	1.30	0.72	-0.78	6.36E-04	1.04E-04	-3.84	3.186E-04
Solution 3	1.40	0.82	-0.68	5.97E-04	1.39E-04	-3.71	4.317E-04

Feedback control experiments- standard setup

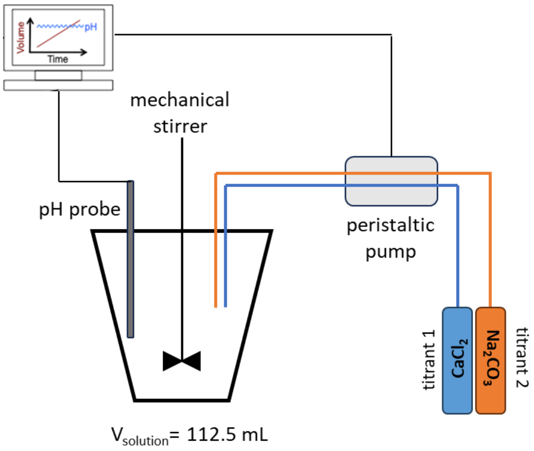

Figure S2: Illustration of the standard feedback control setup. The setup consists of a cone-shaped reaction vessel and the volume of the reaction solution is 112.5 mL. A mechanical stirrer is used to ensure solution mixing. A pH probe with 0.01-unit resolution is inserted in the reaction vessel to measure and record solution pH. Two titrant tubings are introduced into the reaction vessel from the same inlet for addition of titrant solutions of $CaCl_2$ and Na_2CO_3 via a peristaltic pump, which is controlled by the detected pH changes due to calcium carbonate precipitation.

Feedback control experiments- modified setup

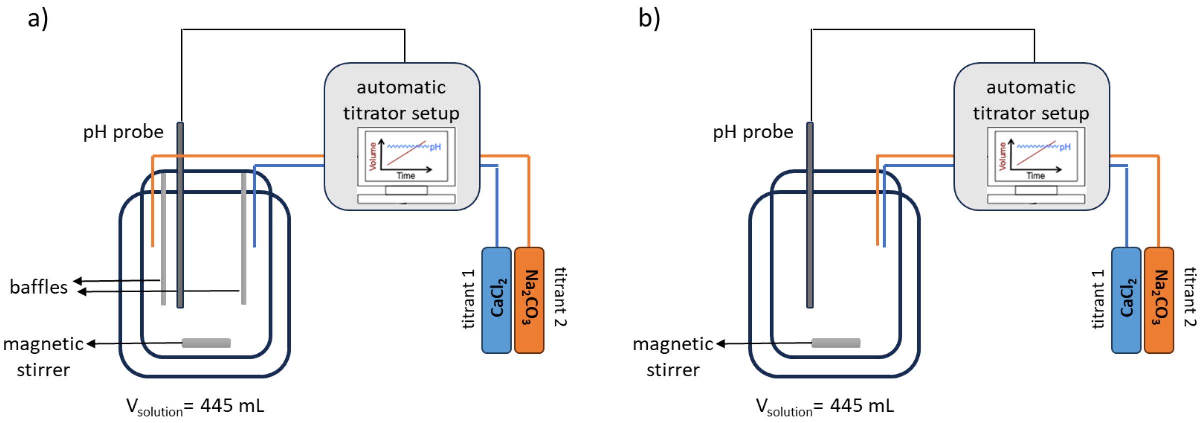

Figure S3: Illustration of the modified feedback control setup operated at (a) sufficient mixing conditions, and (b) insufficient mixing conditions. The setup consists of a jacketed flat bottom reaction vessel and the volume of the reaction solution is 445 mL. A pH probe with 0.002-unit resolution is inserted in the reaction vessel to measure and record solution pH. Two titrant tubings are introduced into the reaction vessel for addition of titrant solutions of $CaCl_2$ and Na_2CO_3 via an automatic titrator, which is controlled by the detected pH changes due to calcium carbonate precipitation. In (a) a magnetic stirrer operating at 300 rpm and two baffles are used to ensure sufficient solution mixing. The titrant tubings are introduced from opposite inlets. In (b) a magnetic stirrer operating at 200 rpm is used and baffles are removed. The titrant tubings are introduced from the same inlet. Adapted from reference [5].

Reference study of $CaCO_3$ precipitation in standard feedback control setup

No nanosheets or seeds were added to this experiment. Supersaturated solutions at $\sigma_{calcite}$ = 1.2 were prepared and samples were taken after 15 min (before any titrant addition), after 3 and 5 hours by placing a sample droplet (approx. 3 uL) on a Si wafer and letting it dry. At the end of the experiment, the solution was vacuum filtered (filter pore size 200 nm), and no particles could be detected on the filter.

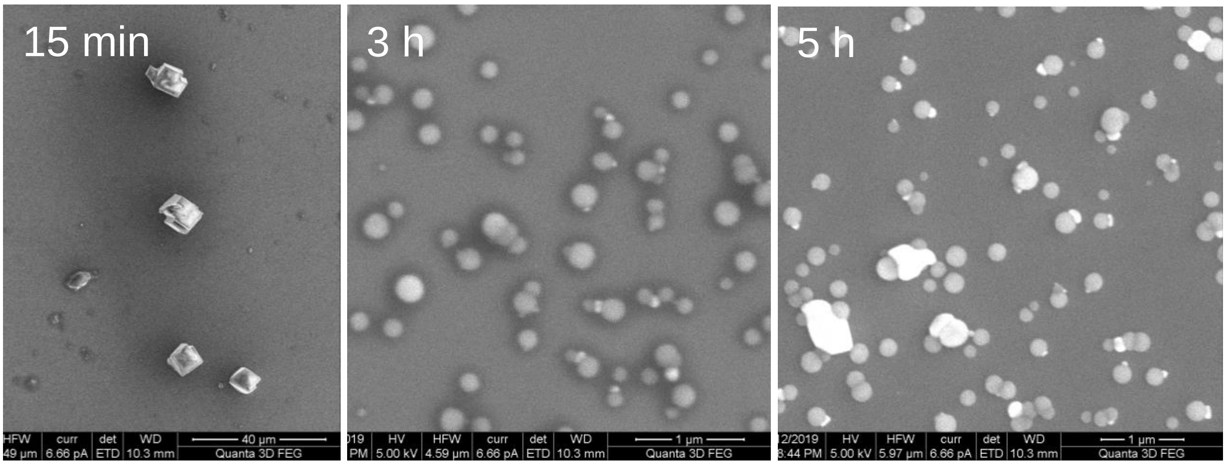

Figure S4: SEM images of samples collected from a standard feedback control experiment after 15 min, 3h and 5h.

For the 15 min sample, calcite crystals of approx. 6 µm precipitated (Fig.S6). Since no calcite crystals were found on the 200 nm filter at the end of the experiment, these crystals must be an effect of sample drying. No calcite was seen for samples taken at 3 h and 5 h. Morphology is instead spherical here with smaller size scale. The polymorph was not investigated, yet, observed precipitates are believed to be drying artifacts due to lack of precipitation observed when solution was filtered.

Theoretical calculations of the amount of ACC that can form during drying was made by assuming evaporation of a 3 µL sampled solution at $\sigma_{calcite}$ = 1.2 to 1 µL. Supersaturation with respect to ACC upon evaporation was calculated to be σ=0.499 and the theoretical amount of ACC that can precipitate was calculated to be 4.61E-04 mol/L.

Reference study of $CaCO_3$ precipitation to investigate drying artifacts

In order to verify the source of precipitation observed on nanosheets and on wafer, further investigations were carried out by drop-casting small aliquots of supersaturated mineralization solution ($\sigma_{calcite}$ = 1.2 and pH 9.0) *with* and *without* nanosheets on silicon wafers and let dry. In the absence of sheets, SEM images showed precipitation of $CaCO_3$ particles with typical vaterite and calcite morphology (Fig. S5). Drying of the supersaturated mineralization solution that contained 1 mM of nanosheets on wafer similarly resulted in observations of precipitation, yet, the nanosheets were not heavily decorated as observed for crystallization experiments conducted with the standard feedback-controlled setup (Fig. S6). Both large crystals with typical vaterite and calcite morphology, and small ACC-like particles were observed on the sheets.

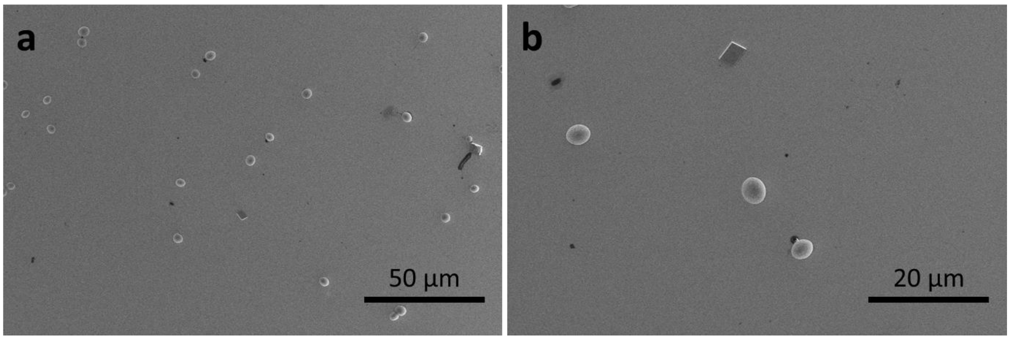

Figure S5: (a,b) SEM images of particles formed on wafer by drying aliquots of supersaturated mineralization solution ($\sigma_{calcite}$ = 1.2 and pH 9.0) without nanosheets.

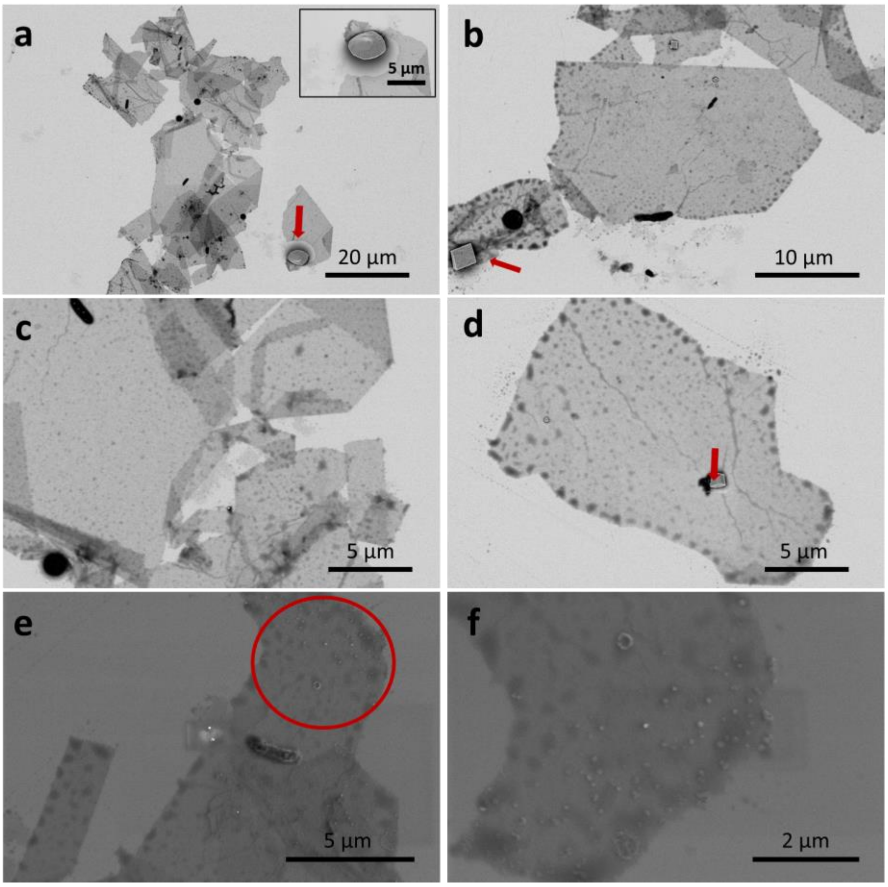

Figure S6: (a-f) SEM images of samples formed on wafer by drying aliquots of supersaturated mineralization solution ($\sigma_{calcite}$ = 1.2 and pH 9.0) with 1 mM of nanosheets. (a,b,d) Large crystals with typical vaterite and calcite morphology were observed on the sheets as shown by red arrows. Nanosheets were not heavily mineralized upon drying. e) A region marked with the red circle shows ACC-like particles sparsely localized on the nanosheet surface, f) Image shows the marked region in (e) with a higher magnification.

Fragmentation of nanosheets

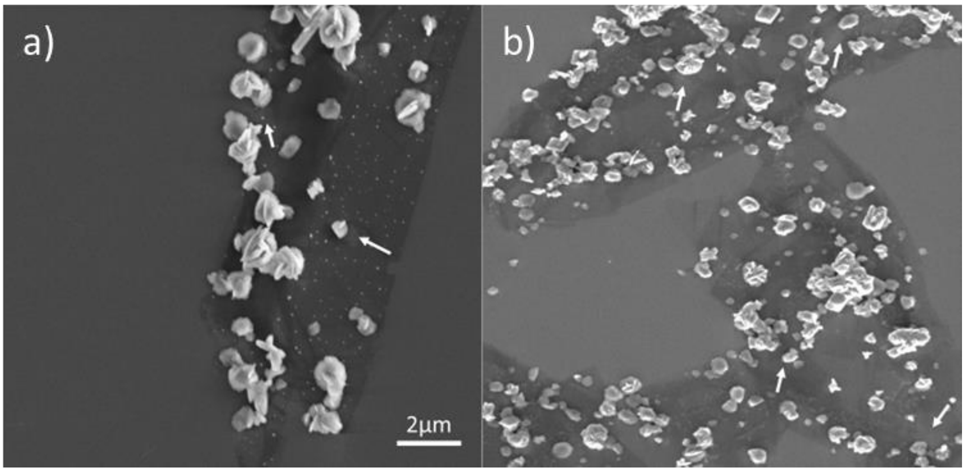

Figure S7: (a-b) SEM images of nanosheets mineralized in batch experiments. Arrows show the fragmentation of nanosheets on wafers due to particles formed on the side facing down.

Peptoid nanosheets

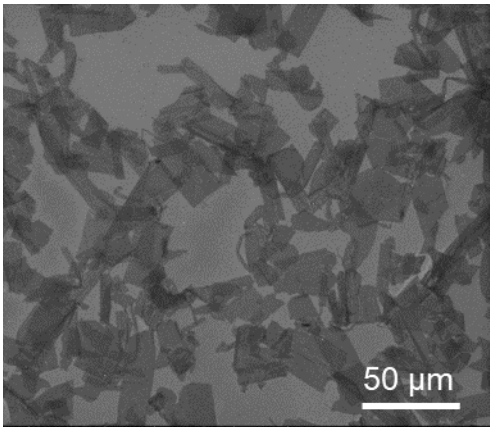

Figure S8: SEM image of peptoid nanosheets prior to mineralization.

Electron dispersive X-ray spectroscopy (EDXS) of mineralized nanosheets in standard feedback control setup

Scanning electron microscopy with energy dispersive X-ray diffraction show an elemental composition of 11% Ca, 18% C, 19% O, 1% Na and 51% Si from the substrate wafer. The analyzed nanosheets were extracted after 4 h.

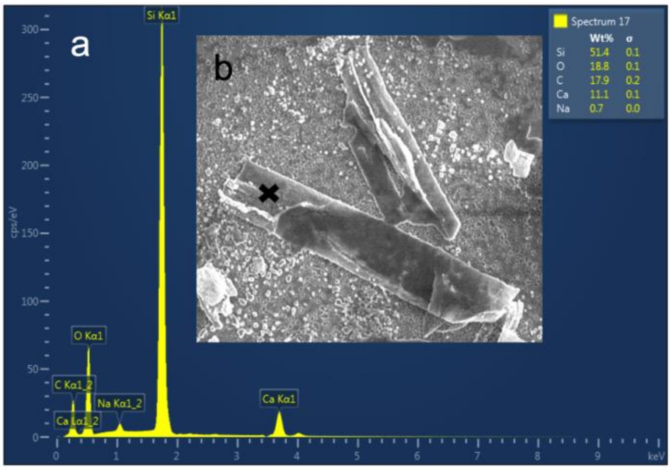

Figure S9: a) EDXS of mineralized nanosheet in standard feedback control setup. The sheet is rich in C, O, and Ca with traces of Na and we also measure Si from the substrate. b) The cross in the image shows where the EDXS spectrum is measured.

Aging of nanosheets mineralized in standard feedback control setup

An aliquot of the reaction solution with nanosheets were kept for 10 days to investigate the effects of aging on the mineral content. Aging at room temperature induced recrystallization of ACC to vaterite.

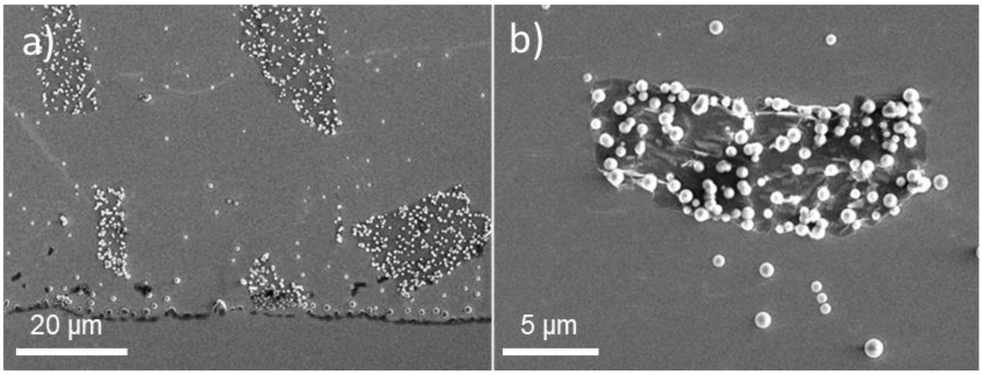

Figure S10: SEM images of a) sample withdrawn after aging of nanosheets mineralized in standard feedback control setup showing decorated nanosheets and b) a higher magnification image focusing on a single sheet with vaterite mineralization.

Reference study of $CaCO_3$ precipitation in modified feedback control setup

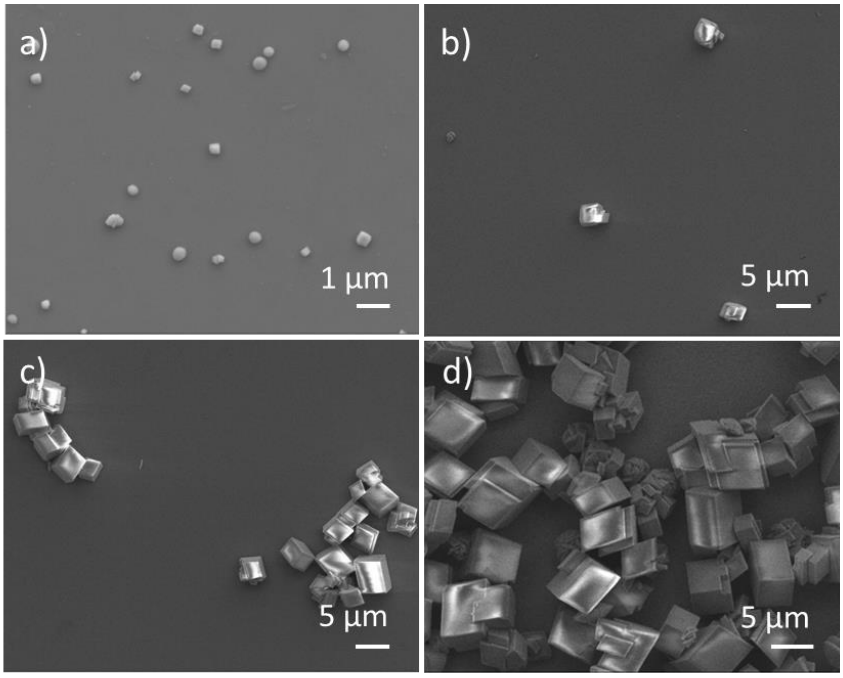

Figure S11: SEM images of samples collected from modified feedback control experiments under good mixing conditions and without peptoid nanosheets at varying time points, a) 50 min, b) 210 min, c) 410 min, d) 425 min.

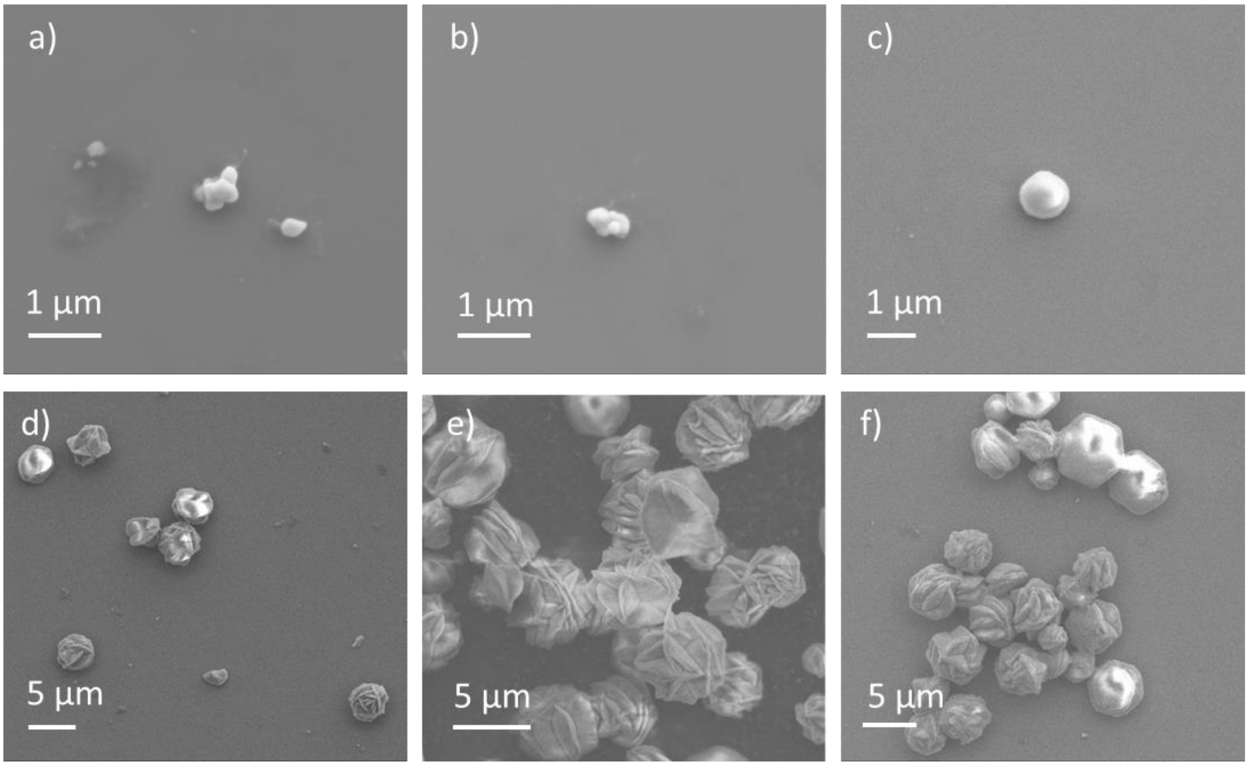

Figure S12: SEM images of samples collected from modified feedback control experiments under poor mixing conditions and 200 mM titrant concentration without peptoid nanosheets at varying time points, a-c) 20 min, d) 35 min, e) 45 min, f) 65 min.

References

1. Kudirka, R.; Tran, H.; Sanii, B.; Nam, K. T.; Choi, P. H.; Venkateswaran, N.; Chen, R.; Whitelam, S.; Zuckermann, R. N., Folding of a single-chain, information-rich polypeptoid sequence into a highly ordered nanosheet. *Biopolymers* **2011,** *96* (5), 586-95.
2. Olivier, G. K.; Cho, A.; Sanii, B.; Connolly, M. D.; Tran, H.; Zuckermann, R. N., Antibody-Mimetic Peptoid Nanosheets for Molecular Recognition. *ACS Nano* **2013,** *7* (10), 9276-9286.
3. Plummer, L. N.; Busenberg, E., The solubilities of calcite, aragonite and vaterite in CO2-H2O solutions between 0 and 90°C, and an evaluation of the aqueous model for the system CaCO3-CO2-H2O. *Geochim. Cosmochim. Acta* **1982,** *46* (6), 1011-1040.
4. Brečević, L.; Nielsen, A. E., Solubility of amorphous calcium carbonate. *J. Cryst. Growth* **1989,** *98* (3), 504-510.
5. Beck, R.; Seiersten, M.; Andreassen, J. P., The constant composition method for crystallization of calcium carbonate at constant supersaturation. *J. Cryst. Growth* **2013,** *380*, 187-196.
